# The additive effect of vitamin K supplementation and bisphosphonate on fracture risk in post-menopausal osteoporosis: a randomised placebo controlled trial

**DOI:** 10.1007/s11657-023-01288-w

**Published:** 2023-06-20

**Authors:** Amelia E. Moore, Dwight Dulnoan, Kieran Voong, Salma Ayis, Anastasios Mangelis, Renata Gorska, Dominic J. Harrington, Jonathan C. Y. Tang, William D. Fraser, Geeta Hampson

**Affiliations:** 1 Osteoporosis Unit, Guy’s Hospital, London, UK; 2 Nutristasis Unit, Synnovis Analytics, St Thomas’ Hospital, London, UK; 3 School of Life Course and Population Sciences, Faculty of Life Sciences & Medicine, King’s College London, Guy’s Campus, London, UK; 4 Norwich Medical School, University of East Anglia, Norwich, UK; 5 Depts of Endocrinology and Clinical Biochemistry Norfolk and Norwich University Hospitals Trust, Norwich, UK; 6 Department of Chemical Pathology and Metabolic Medicine, St Thomas’ Hospital, Lambeth Palace Road, London, SE1 7EH UK; 7 Metabolic Bone Clinic, Department of Diabetes and Endocrinology, St Thomas’ Hospital, London, UK

**Keywords:** Vitamin K, Post menopausal osteoporosis, Bisphosphonate, Hip geometry

## Abstract

***Summary*:**

This study assessed whether vitamin K, given with oral bisphosphonate, calcium and/or vitamin D has an additive effect on fracture risk in post-menopausal women with osteoporosis. No difference in bone density or bone turnover was observed although vitamin K_1_ supplementation led to a modest effect on parameters of hip geometry.

**Purpose:**

Some clinical studies have suggested that vitamin K prevents bone loss and may improve fracture risk. The aim was to assess whether vitamin K supplementation has an additive effect on bone mineral density (BMD), hip geometry and bone turnover markers (BTMs) in post-menopausal women with osteoporosis (PMO) and sub-optimum vitamin K status receiving bisphosphonate, calcium and/or vitamin D treatment.

**Methods:**

We conducted a trial in 105 women aged 68.7[12.3] years with PMO and serum vitamin K_1_ ≤ 0.4 µg/L. They were randomised to 3 treatment arms; vitamin K_1_ (1 mg/day) arm, vitamin K_2_ arm (MK-4; 45 mg/day) or placebo for 18 months. They were on oral bisphosphonate and calcium and/or vitamin D. We measured BMD by DXA, hip geometry parameters using hip structural analysis (HSA) software and BTMs. Vitamin K_1_ or MK-4 supplementation was each compared to placebo. Intention to treat (ITT) and per protocol (PP) analyses were performed.

**Results:**

Changes in BMD at the total hip, femoral neck and lumbar spine and BTMs; CTX and P1NP did not differ significantly following either K_1_ or MK-4 supplementation compared to placebo. Following PP analysis and correction for covariates, there were significant differences in some of the HSA parameters at the intertrochanter (IT) and femoral shaft (FS): IT endocortical diameter (ED) (% change placebo:1.5 [4.1], K_1_ arm: -1.02 [5.07], *p* = *0.04*), FS subperiosteal/outer diameter (OD) (placebo: 1.78 [5.3], K_1_ arm: 0.46 [2.23] *p* = *0.04*), FS cross sectional area (CSA) (placebo:1.47 [4.09],K_1_ arm: -1.02[5.07], *p* = *0.03*).

**Conclusion:**

The addition of vitamin K_1_ to oral bisphosphonate with calcium and/or vitamin D treatment in PMO has a modest effect on parameters of hip geometry. Further confirmatory studies are needed.

**Trial registration:**

The study was registered at Clinicaltrial.gov:NCT01232647.

**Supplementary Information:**

The online version contains supplementary material available at 10.1007/s11657-023-01288-w.

## Introduction

Osteoporosis is the most common bone disease and affects one in two postmenopausal women. Osteoporosis-related fractures cause severe pain and disability, loss of independence and increased mortality particularly in those who sustain a hip fracture. This places an enormous burden on healthcare resources in excess of £4.7 billion annually in the United Kingdom (UK) [1]. Improving bone strength and reducing fracture risk in individuals with osteoporosis is therefore of crucial importance. Oral bisphosphonates (alendronate or risedronate) are often first line treatments in post-menopausal osteoporosis (PMO). They improve bone mineral density (BMD) and reduce fracture risk [1]. Fracture risk reduction in differing skeletal locations with these drugs is in the region of 40–60%, which leaves room for the addition of other agents to improve treatment efficacy in this high-risk population [2, 3].

Several epidemiological and cross-sectional studies have highlighted the importance of vitamin K (best known for its effects on blood clotting) for bone health. The two main forms of vitamin K; vitamin K_1_ (phylloquinone) and K_2_ (the menaquinones series; MKs) act as co-factors for the enzyme γ-glutamyl carboxylase which catalyses the conversion of glutamate residues (Glu) to γ-carboxyglutamate residues (Gla). This process confers biological activity to the vitamin K-dependent proteins (VKDPs) [4]. The VKDPs include several proteins present in bone. Amongst those, osteocalcin (OC) and Matrix Gla Protein (MGP) are the most studied. OC is expressed by osteoblasts and involved in the mineralisation of bone matrix through its calcium-binding properties. MGP is an inhibitor of vascular calcification and also has a possible role in normal bone metabolism [5, 6].

Daily adequate intake of vitamin K_1_ to maintain nutritional adequacy is estimated as 90 µg for women and 120 µg for men [7]. Whilst this may be sufficient for blood coagulation, higher intake may be needed for its skeletal effects. Low vitamin K status and low dietary intake have been shown to be associated with low BMD and increased fractures, particularly hip fractures as described in a meta-analysis of over 80, 000 participants [8]. Women with a daily intake of vitamin K of > 109 µg/day had a 30% reduction in the risk of hip fracture compared to those with an intake < 109 µg (RR: 0.70; 95% CI: 0.53, 0.93) [9]. Vitamin K has also been shown to improve other indices of bone strength such as hip structural and mechanical parameters independently of BMD [10]. Data from previous studies [11, 12] would suggest that a threshold of serum vitamin K_1_ in excess of 0.7 µg/L may be considered as ‘sufficient’ for bone health as this would biologically enable optimum carboxylation of the vitamin K dependent binding proteins (VKDPs) present in bone. In situations of low vitamin K, there is preferential carboxylation of hepatic VKDPs involved in coagulation instead of the extra-hepatic VKDPs.

Clinical trials of vitamin K supplementation have however been inconclusive and data from some of the early work has been questioned. In a more recent meta-analysis of intervention trials, excluding the trials where concerns were raised, in post-menopausal or osteoporotic patients, a reduction in clinical fractures for vitamin K compared to controls was seen (OR, 0.72, 95% CI 0.55 to 0.95) with no difference in vertebral fractures or BMD [13]. A 2 year randomised controlled trial of high dose vitamin K_1_ (5 mg/day), extended for a further 2 years also showed no change in BMD but a significant reduction in clinical fractures after 4 years although the study was not powered to examine fractures [14]. Because of the heterogeneity of these trials in terms of study design, length of treatment and variations in treatment regimes, participants characteristics, it is difficult to translate the findings into clinical practice. Another limitation is that the vitamin K status of the participants was not known in many trials. It is unclear whether a larger effect may have been demonstrated in vitamin K insufficient post-menopausal women with osteoporosis. However, despite the conflicting results, evidence so far may indicate that some population groups such as subjects with osteoporosis and/or low vitamin K status may be more at risk of fracture and the effect of vitamin K supplementation in this high-risk group should be studied. The use of vitamin K in the prevention of bone loss and/or fractures in high-risk post-menopausal women with osteoporosis in whom vitamin K nutritional status may be inadequate should therefore not be dismissed as there have been very few studies in this population.

Whilst we do not advocate using vitamin K_1_ and K_2_ (MK-4) as single first-line agents in this population, there are interesting preliminary data which show that combined treatment with a bisphosphonate and vitamin K, at least vitamin K_2_ (MK-4), appears to be more effective than single-treatment with a bisphosphonate [15, 16].

We hypothesized that the addition of vitamin K in the form of either K_1_ or K_2_ (MK-4) to standard conventional treatment with oral bisphosphonate and calcium and/or vitamin D supplements in women with PMO who have sub-optimum vitamin K status as defined by serum vitamin K_1_ concentration below the median for the cohort, would provide further benefits to their bone health and improve fracture risk. The aims of this randomised controlled trial were to investigate changes in 1. BMD at the total hip, femoral neck and lumbar spine, 2. Biomarkers of bone formation and resorption and 3. Hip structural parameters following supplementation with vitamin K_1_ (1 mg/day) or K_2_ (MK-4, 45 mg/day) compared to placebo for 18 months in vitamin K deplete post-menopausal women with osteoporosis who are already on standard treatment with oral bisphosphonate and combined calcium/vitamin D or vitamin D alone.

## Subjects and methods

### Participants and study design

The vitamin K in osteoporosis trial was an 18 month randomised controlled trial (RCT) conducted in 105 ambulatory post-menopausal women with osteoporosis (PMO) aged between 55–85 years. Participants who had been on oral bisphosphonate and/or calcium/vitamin D supplements for fracture prevention for at least 12 months were invited to take part in the study. Exclusion criteria included secondary osteoporosis such as inflammatory joint or bowel disorders, current glucocorticoid therapy, chronic kidney disease stage 4 and 5, malabsorption, untreated endocrine disorders (primary hyperparathyroidism, hyperthyroidism) or current or past treatment (< 12 months) with other osteoporosis drugs e.g. teriparatide, denosumab, hormone replacement therapy or selective oestrogen receptor modulators and treatment with vitamin K antagonists; warfarin. Participants were recruited from April 2015 to September 2018 from the metabolic bone clinics, the osteoporosis unit at Guy’s Hospital, primary care or through community advertising and public engagement activities as previously described [11].

Ethical approval was obtained by the National Research Ethics Service (NRES Committee London—Westminster), approval number10/H0802/72. The trial was conducted according to the protocol and in compliance with the principles of the Declaration of Helsinki, the principles of Good Clinical Practice (GCP) and in accordance with Medicines for Human Use (Clinical Trials) Regulations. The trial protocol and substantial amendments were reviewed and approved by the United Kingdom (UK) Medicines and Healthcare products Regulatory Agency (MHRA). The trial was registered with the European Union Drug Regulating Authorities Clinical Trials Database (EudraCT) (registration number 2010–02258712) and on the ClinicalTrials.gov registry (ID; NCT01232647).

The trial was designed in 2 stages. The first stage was a pre-screening study of up to 374 with PMO on oral bisphosphonate to assess their serum vitamin K_1_ (phylloquinone) concentration. Serum vitamin K_1_ was measured after at least a 2 h fast and those with low serum vitamin K_1_ concentration as described below were invited to take part in the RCT. Serum vitamin K_1_ concentration of ≤ 0.3 µg/L was the initial entry criteria defined as twice the lower limit of the reference range of our laboratory. After review of recruitment rate and data, this was changed to ≤ 0.35 µg/L (twice the lower limit of the fasting range) to improve recruitment and later to ≤ 0.40 µg/L as this value was below the median of the population screened at the time (n = 320). The physiological rationale was based partly on our findings that serum vitamin K_1_ concentrations above 0.65 µg/L was associated with fracture risk reduction [11]. We also found that serum Vitamin K_1_ of greater than 0.7 µg/L led to undetectable concentrations of undercarboxylated MGP. All participants gave written informed consent. Those who met the study entry criteria were invited to take part in the randomised doubleblind placebo controlled trial of vitamin K_1_ or vitamin K_2_ (MK-4: menatretrenone). Following informed written consent, 105 participants were randomised to 3 groups; placebo, vitamin K_1_ arm (1 mg/day) or vitamin K_2_ arm (MK-4 45 mg/day in 3 divided doses). A similar design and dose of vitamin K_1_ and MK-4 as previously described was adopted [17]. In addition, 1 mg/day dose of vitamin K_1_ has been shown to maximally carboxylate osteocalcin [18]. Participants were randomly assigned to one of 3 treatment arms. As the study was double-blind, we used different matching placebos for K_1_ and MK-4. Subjects were randomised as follows; vitamin K_1_ (1 mg/day) plus placebo matching MK-4 three times/day or placebo matching vitamin K_1_ plus MK-4 (15 mg three times daily) or placebo vitamin K_1_ and placebo MK-4 three times daily.

All 3 groups were advised to continue with their standard treatment of oral bisphosphonate and vitamin D (800 IU/day) if their dietary calcium intake was satisfactory (> 1 g/day) or combined calcium/vitamin D supplements (1.0 g calcium and 800 IU vitamin D/day) if their dietary calcium intake was low (< 500 mg/day). A detailed clinical history and examination were done at baseline. BMD and routine blood tests were measured at baseline and at 18 months. Bone turnover markers and serum vitamin K_1_ were also measured at baseline and at each study visit at 3, 6, 12 and 18 months. Adverse events were documented at each study visit. Participants were asked to take their vitamin K supplements following or soon after their meals. No special dietary or lifestyle requirements or changes were needed.

**Fig. 1:**
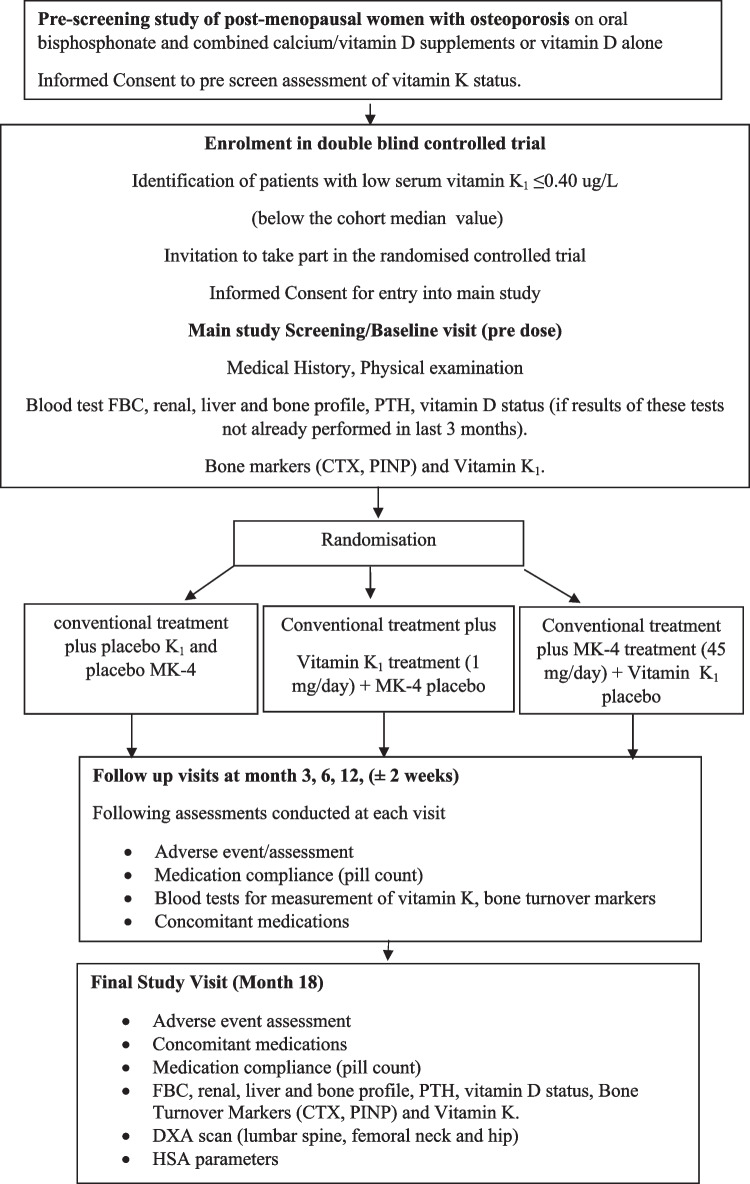
Flowchart illustrating the study protocol

Primary outcomes were changes in BMD at the lumbar spine (LS), femoral neck (FN) and total hip (TH) at 18 months between the 2 treatment arms (vitamin K_1_ arm and vitamin K_2_ (MK-4) arm) compared to placebo. Secondary outcomes were differences in bone turnover as assessed by BTMs and serum vitamin K_1_ measured at baseline, 3, 6, 12 and 18 months. Additional secondary objectives included parameters of hip geometry at three other hip sites, namely narrow neck of femur (NN), intertrochanteric (IT) and femoral shaft (FS) derived using the HSA software and the DXA scan images acquired during the BMD measurements of the hip at baseline and 18-months. The study design is summarised in Fig. 1; the trial flowchart.

### Study medications

The study medications were vitamin K_1_ given as 1 tablet containing 1.0 mg and menatetrenone (vitamin K_2_) or MK-4 45 mg given as 3 tablets containing 15 mgs. These tablets as well as the respective matching placebo tablets were manufactured from pharmaceutical grade phylloquinone (K_1_) and menatretrenone (MK-4) and QP released as defined in the UK Clinical Trial Regulations and supplied in blister packs labelled by the manufacturers (90 tablets of vitamin K_1_ (or placebo) and 90 tablets per blister pack of MK-4 (or placebo) by Tiofarma B.V, 3260 BB Oud-Beijerland, Netherlands. The blister packs were stored and dispensed by the Guy’s Hospital Clinical Trials pharmacy. Block randomisation was done by means of a validated computerised system, designed by the Clinical Trials Unit at Kings College London. The study investigators and participants were blinded to the study allocation as were the staff performing the DXA scan analyses and the bone turnover markers analyses. Study medications was provided at each study visit. Compliance was estimated at each study visit on tablet count from the returned blister packs.

### Dual energy X-ray absorptiometry (DXA) and hip structure analysis (HSA)

DXA scans to assess BMD at the LS, TH and FN were carried out using the Hologic Discovery scanner (Hologic, Inc. Bedford, MA, USA). The coefficient of variation (CV) for positioning and BMD measurement at the spine and total hip was 1.0% and 1.2%, respectively. BMD was measured at baseline (within 3 months of study entry) and 18 months later using the same scanner for each participant throughout the study.

Hip geometry analyses at baseline and 18 months were done using the HSA program and the femur image within the DXA analysis software [19]. Regions of interest and femur positioning were checked by a certified DXA technologist who performed the HSA analyses on all participants using the standardised HSA analysis protocol. Analysis sites included the NN across the narrowest diameter of the femoral neck, the IT along the bisector of the neck shaft angle and FS which is 2 cm distal to the midpoint of the lesser trochanter. The program derived the following measurements at the NN, IT and FS: 1) subperiosteal (outer) width or diameter (OD) 2) endocortical (inner) width or diameter (ED) 3) cross-sectional area (CSA) which provides an index of resistance to axial forces, 4) estimated cortical thickness (Co Th), 5) cross-sectional moment of inertia (CSMI) which gives an estimate of resistance to bending forces and structural rigidity, 6) section modulus (Z) which is an indicator of bending strength, 7) buckling ratio (BR) which provides an estimate of susceptibility to local cortical buckling under compressive loads, 8) neck shaft angle (NSA), and 9) hip axis length (HAL).

### Laboratory measurements

All blood samples were obtained after at least a 2 h fast. Samples for routine laboratory measurements were taken at baseline and at 18 months. Biochemical tests included renal/liver/bone profile, parathyroid hormone (PTH) and were done by standard laboratory methods on the Roche automated analysers (Roche diagnostics Limited, West Sussex, UK). eGFR was calculated from serum creatinine using the Modification of Diet in Renal disease formula. Vitamin D status was determined by measurement of 25-hydroxy vitamin D (25(OH)D) using an immunoassay on the automated Abbott Architect analyser (Abbott Laboratories, Abbott Park, Illinois, USA). PTH assay CVs were < 5% at PTH concentrations of 41 and 105 ng/L. 25(OH)vitamin D assay CVs ranged between 5.0 and 10.7% at serum 25 (OH)vitamin D concentrations between 25 and 85 nmol/L. The reference ranges for PTH is 10–65 ng/L, serum albumin: 40–52 g/L, serum creatinine: 45-84 µmol/L. Serum 25(OH)vitamin D > 50 nmol/L is considered sufficient, 25–50 nmol/L: insufficient and < 25 nmol/L: deficient.

Blood samples for serum vitamin K_1_ (phylloquinone) and the BTMs were obtained at baseline and at each study visit (3, 6, 12 and 18 months). Serum vitamin K_1_ was measured by liquid chromatography tandem mass spectroscopy (LCMSMS). The assay reference range was 0.17–0.68 µg/L (0.38–1.51 nmol/L) in fasting adults and 0.15–0.55 µg/L (0.33–3.44 nmol/L) in non-fasting state. Assay CV was < 8%. The BTMs; total procollagen 1 N-terminal propeptide (P1NP); marker of bone formation and beta C-terminal telopeptide (CTX); marker of bone resorption were measured by electrochemiluminescence immunoassay (ECLIA) on a COBAS e601 analyser (Roche Diagnostics, Mannheim, Germany) following the manufacturer’s instructions. Inter-assay coefficient of variation (CV) for PINP was < 2.3% across the analytical range of 5–1200 ng/mL with a sensitivity of 5 ng/mL Inter-assay (CV) for CTX was < 6.8% across the analytical range of 0.01‑6.00 ng/mL, with a sensitivity of 0.01 ng/mL.

### Safety

All participants were monitored at regular intervals (3, 6, 12 and 18 months) and adverse events (AEs) were recorded at each visit.

### Statistical analysis

The primary efficacy endpoint was changes in BMD at the TH, FN and LS at 18 months compared to baseline. The sample size was based on a difference between the control group and treatment groups of a change in BMD of 3% (clinically significant) at the hip with an SD of 3%. Assuming a 10% non-compliance rate, 28 patients in each arm will provide 95% power to detect a change in the primary outcome at the 5% significance level. To make allowance for the unknown distribution of the primary outcome, we increased the sample size by a further 15% to 32 per group. Further accounting for a drop-out rate of 10% consistent with previous trials, 35 patients were recruited in each group for a total study population of 105. The secondary end points included changes in BTMs and HSA parameters.

Statistical analyses were done according to the statistical analysis plan using R Core Team Version 4.0 [20]. Changes in BMD were analysed using ANCOVA and Bonferroni test, adjusting for baseline values based on the intention to treat (ITT) and per protocol (PP) population. The model was checked for independence of residuals, distribution of residuals, equal variance of residuals, distribution of random effects (as appropriate), and extreme outliers and points with high leverage. Values are reported with SD or 95% confidence intervals. The ‘*p*’ value used to determine significance for the primary outcome was set at p ≤ 0.0167 in consideration of the multiple comparisons (Bonferroni adjustment; 0.05/3). Percentage changes in BMD were also derived and reported as mean (SD).

Changes in BTMs as secondary efficacy endpoints were analysed using repeated measures ANCOVA adjusted for baseline based on the intention to treat (ITT) and per protocol (PP) population. Bonferroni test was used for group comparisons. Data for the BTMs were logarithmic transformed to achieve normal distribution. Additional secondary outcome measures included changes in the HSA parameters at 18 months compared to baseline. Differences in HSA were tested by ANCOVA with Bonferroni test (adjustment for multiple comparisons) as for the primary outcome. A *‘p’* value of < 0.05 was considered significant for the secondary outcomes.

Further exploratory analyses were performed including multivariate models correcting for co-variates including age, BMI, baseline serum vitamin K_1_ concentration, duration of treatment with bisphosphonate and type of bisphosphonate (alendronate or risedronate). Results are expressed as mean (SD) or 95% CI. Level of significance was set at < 0.05 in the exploratory analyses.

## Results

### Study subjects

**Fig. 2:**
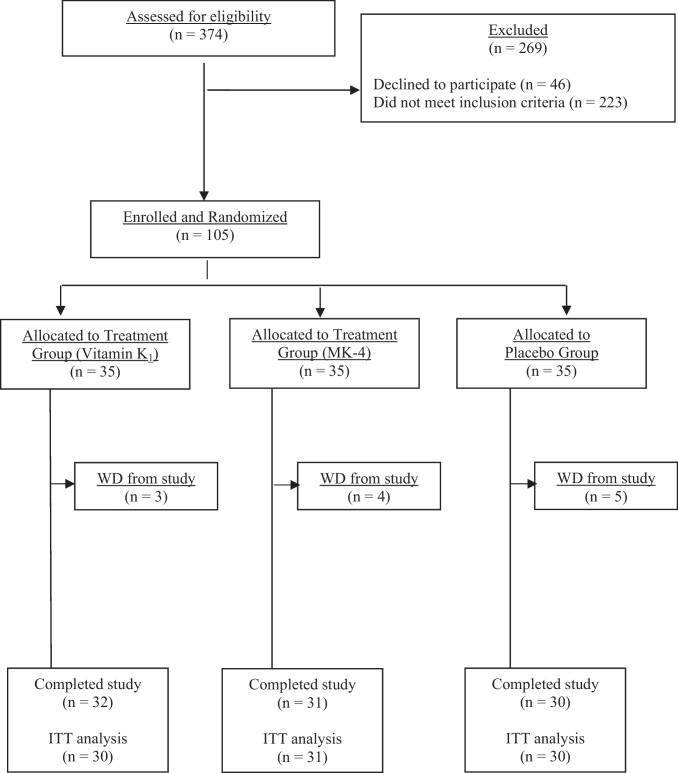
CONSORT flowchart, WD: withdrew from study, ITT: intention to treat

374 community-dwelling ambulant post-menopausal women aged (mean [SD]) 68.7[12.3] years with PMO on treatment with oral bisphosphonates were recruited to the first stage of the study (pre-screen study). They were recruited from the metabolic bone clinics in secondary care (*n* = 202), osteoporosis unit (*n* = 71) at Guy’s Hospital, primary care (*n* = 14) or through community advertising and public engagement activities (*n* = 87). All participants were seen in the Osteoporosis Unit and informed consent was obtained. Following the pre-screen study, 105 women who met the study entry criteria for the randomised controlled trial were invited consecutively to participate in the second stage of the study. Treatment duration with bisphosphonate prior to study entry was mean [SD]; 3.1[2] years. Sixty five women had sustained a previous fragility fracture (62%). Nine participants were on risedronate 35 mg weekly, the rest were on alendronate 70 mg weekly. Ninety women were taking combined calcium and vitamin supplements (placebo *n* = 29, vitamin K_1_ arm *n* = 31, MK-4 arm *n* = 30). The rest were on vitamin D supplement only (placebo *n* = 6, vitamin K1 arm *n* = 4, MK-4 arm *n* = 5). A CONSORT diagram demonstrating the flow of participants through the trial is shown in Fig. 2. Of the 105 women who entered the RCT, 12 withdrew because of the following reasons; lost to follow up or failed to attend subsequent study visits or other commitments outside the study (*n* = 3), stopped oral bisphosphonate (*n* = 1) or changed to other osteoporosis medications (*n* = 1), due to pill burden (*n* = 2), adverse events (AEs) including dizziness (*n* = 1), diarrhoea/reflux (*n* = 1), surgical procedure (*n* = 1), hip fracture following a riding accident (*n* = 1), new diagnosis of primary hyperparathyroidism (*n* = 1). Ninety three women completed the study. Overall compliance with the study medications did not differ between the 3 arms during the trial and at each visit. Adherence was 99.3% in the vitamin K_1_ arm, 96.3% in the MK4 arm and 97% in the placebo arm. Ninety one women were included in the ITT analysis of DXA scan measurements as there were analytical and technical errors in 2 participants (neck of femur (*n* = 1) and lumbar spine (*n* = 1). Seven patients were excluded from the per protocol (PP) analysis because compliance with the study medications (< 70%) *n* = 3), protocol deviation due to Covid-19 pandemic causing a delay of 5 months to their final visit (*n* = 2), oral glucocorticoid use for 2 months (*n* = 1) and compliance to oral alendronate (verbal reporting) (< 50%) during the trial (*n* = 1). At baseline, there were no significant differences in demographics, BMD and biochemical parameters between the 3 groups as shown in Table 1. Although serum vitamin K_1_ concentration was ≤ 0.4 µg/L in all participants during the pre-screen stage, 36 participants had serum K concentration higher than 0.4 µg/L at the study entry baseline visit (placebo *n* = 13, K_1_ arm *n* = 15 and MK-4 arm *n* = 8).

**Table 1:** Table of baseline demographics, biochemical parameters by arm (before any withdrawals) Values are mean (SD)

Characteristics	Vitamin K_1_ arm *N* = 35	MK-4 arm *N* = 35	Placebo *N* = 35	*p-value*
Age (years)	65 (6)	69 (7)	67 (7)	*0.064*
BMI (kg/m^2^)	24.5 (5.6)	23.4 (2.5)	24.9 (5.6)	*0.9*
Treatment duration with bisphosphonate (years)	3.0(2.2)	3.3(2.0)	3.1(1.7)	*0.9*
Total Hip BMD (g/cm^2^)	0.74 (0.09)	0.76 (0.09)	0.76 (0.10)	*0.3*
Total Hip T score	-1.70 (0.72)	-1.47 (0.73)	-1.49 (0.82)	*0.3*
Femoral neck BMD (g/cm^2^)	0.61 (0.09)	0.64 (0.10)	0.63 (0.09)	*0.4*
Femoral neck T score	-2.15 (0.77)	-1.89 (0.93)	-1.95 (0.85)	*0.4*
Lumbar spine BMD (g/cm^2^)	0.79 (0.10)	0.79 (0.10)	0.77 (0.09)	*0.8*
Lumbar spine T score	-2.27 (0.92)	-2.37 (0.90)	-2.46 (0.82)	*0.8*
Creatinine (µmol/L)	65 (9)	65 (9)	67 (13)	*0.9*
eGFR (ml/min)	81 (14)	81 (16)	79 (15)	*0.9*
Albumin adjusted calcium (mmol/L)	2.39 (0.09)	2.39 (0.10)	2.35 (0.08)	*0.12*
Parathyroid Hormone (PTH) ng/L Ref. range: 10–65 ng/L	38 (13)	37 (12)	42 (19)	*0.6*
25(OH)vitamin D (nmol/L)	80 (27)	83 (29)	79 (25)	*0.9*
Serum Vitamin K _1_ (µg/L)	0.38 (0.23)	0.34 (0.26)	0.45 (0.26)	*0.067*
CTX (µg/L) Ref. range: 0.1–0.5 μg/L	0.20 (0.11)	0.17 (0.08)	0.18 (0.08)	*0.7*
P1NP (µg/L) Ref range: Pre- Menopausal women: 30—78 µg/L Post Menopausal women: 26 -110 µg/L	31 (19)	26 (12)	25 (9)	*0.7*

There were no significant differences between the 3 groups

### Effect of vitamin K1 and MK-4 on bone mineral density (BMD)

There was no significant difference between the vitamin K_1_ or MK-4 arm and placebo arm in LS, FN and TH BMD in either the ITT analysis or the PP analysis. Percentage change in BMD from baseline in the 3 groups is shown in Table 2. The mean [SD] % change in TH BMD following ITT analysis was 0.71% [3.27] in the vitamin K_1_ arm compared to -0.23% [2.35] in the placebo arm *(p* = *0.18).* Adjusting for co-variates in the ANCOVA model did not show any significant differences in treatment effect, although we observed significant interaction between BMI and BMD changes at the FN (ITT: *p* = *0.012* and LS, (PP: *p* = *0.007*).

**Table 2:** There were no significant differences in BMD between the vitamin K_1_ or MK4 arm and placebo arm at the LS, FN and TH BMD. Results are expressed as % change from baseline

DXA (ITT analysis)	Placebo *n* = 30			Vitamin K_1_ *n* = 30			MK-4 *n* = 31			*P value*
mean [SD]	Baseline	18-months	% change	Baseline	18-months	% change	Baseline	18-months	% change	
Lumbar spine (LS) BMD (g/cm^2^)	0.760 (0.085)	0.775 (0.087)	2.11 (5.80)	0.790 (0.101)	0.796 (0.105)	0.59 (3.06)	0.793 (0.100)	0.802 (0.116)	1.00 (4.06)	0.19* *0.78* ^*#*^
Femoral neck (FN) BMD ( (g/cm^2^)	0.619 (0.090)	0.633 (0.095)	1.19 (5.57)	0.617 (0.088)	0.625 (0.089)	1.44 (5.59)	0.643 (0.105)	0.646 (0.109)	0.50 (3.18)	0.83* *0.63* ^*#*^
Total hip (TH) BMD (g/cm^2^)	0.744 (0.092)	0.745 (0.095)	-0.23 (2.35)	0.736 (0.093)	0.742 (0.096)	0.71 (3.27)	0.761 (0.089)	0.761 (0.094)	-0.04 (2.63)	0.18* *0.75* ^*#*^
DXA (PP analysis)	Placebo *n* = 28			Vitamin K1 *n* = 28			MK-4 *n* = 28			*P value*
mean [SD]	Baseline	18-months	% change	Baseline	18-months	% change	Baseline	18-months	% change	
Lumbar spine (LS) BMD	0.765 (0.083)	0.775 (0.090)	1.23 (3.35)	0.789 (0.102)	0.792 (0.105)	0.46 (3.05)	0.790 (0.097)	0.804 (0.112)	1.65 (3.49)	0.61* *0.63* ^*#*^
Femoral neck (FN) BMD	0.625 (0.086)	0.630 (0.095)	0.73 (5.09)	0.618 (0.088)	0.622 (0.092)	0.77 (3.92)	0.649 (0.106)	0.653 (0.109)	0.59 (3.12)	0.48* *0.96* ^*#*^
Total hip (TH) BMD	0.745 (0.094)	0.743 (0.095)	-0.28 (2.38)	0.738 (0.095)	0.740 (0.099)	0.24 (1.84)	0.769 (0.085)	0.768 (0.090)	-0.17 (2.59)	0.26* *0.89* ^*#*^

Table 2A and B represent intention to treat (ITT) and per protocol (PP) analysis respectively, p* vitamin K_1_ v/s placebo, *p*^*#*^ MK-4 v/s placebo

### *Effect of vitamin K*_*1*_* and MK-4 on biochemical markers of bone turnover*

We did not observe any significant differences in plasma CTX or P1NP between the treatment arms compared to placebo as shown in Table 3. However, we observed a significant trend in increases in CTX and P1NP (*p* < 0.001) over time in all 3 arms. No significant effect was observed after adjusting for duration of treatment with bisphosphonate. Similar findings were observed following PP analysis. Serum vitamin K_1_ increased significantly in the vitamin K_1_ treatment arm compared to placebo (Supplementary Table 1).

**Table 3:** % Change in biochemical markers of bone turnover; β C-terminal telopeptide (βCTX) and total procollagen 1 N-terminal propeptide (P1NP) over time (months) following intention to treat (ITT) analysis

CTX							
Time points Months	Placebo *n* = 30	95% CI	Vitamin K_1_ arm *n* = 32	95% CI	MK-4 *n* = 31	95% CI	*P value*
3	5	-13, 24	2	-10, 13	8	-6.4, 22	0.6* *0.6*^*#*^
6	7	-10.0, 24	-6	-18, 5.7	5	-9.1, 20	0.43* *0.95*^*#*^
12	15	-1.9, 32	5	-8.3, 18	22	4.3, 40	0.6* *0.55*^*#*^
18	18	-4.5, 41	16	-5.1, 37	20	4.4, 36	0.84* *0.54*^*#*^
P1NP							
Time points Months	Placebo *n* = 30	95% CI	Vitamin K_1_ arm *n* = 32	95% CI	MK4 *n* = 31	95% CI	*P value*
3	0	-6.7, 6.9	2	-7.0, 10	7	-4.7, 19	0.3* *0,4*^*#*^
6	11	-0.93, 23	-4	-14, 6.9	8	-4.8, 22	0.3* *0.7*^*#*^
12	29	1.1, 57	5	-9.4, 20	13	0.65, 26	0.13* *0.38*^*#*^
18	22	11, 32	7	-6.4, 21	40	-1.2, 81	0.42* *0.8*^*#*^

No significant differences were seen between vitamin K_1_ arm, MK-4 arm and placebo. Results are expressed as mean and 95% confidence interval (CI) p* vitamin K_1_ v/s placebo, *p*# MK-4 v/s placebo

### *Effect of vitamin K*_*1*_* and MK-4 on hip structural parameters (HSA)*

**Fig. 3:**
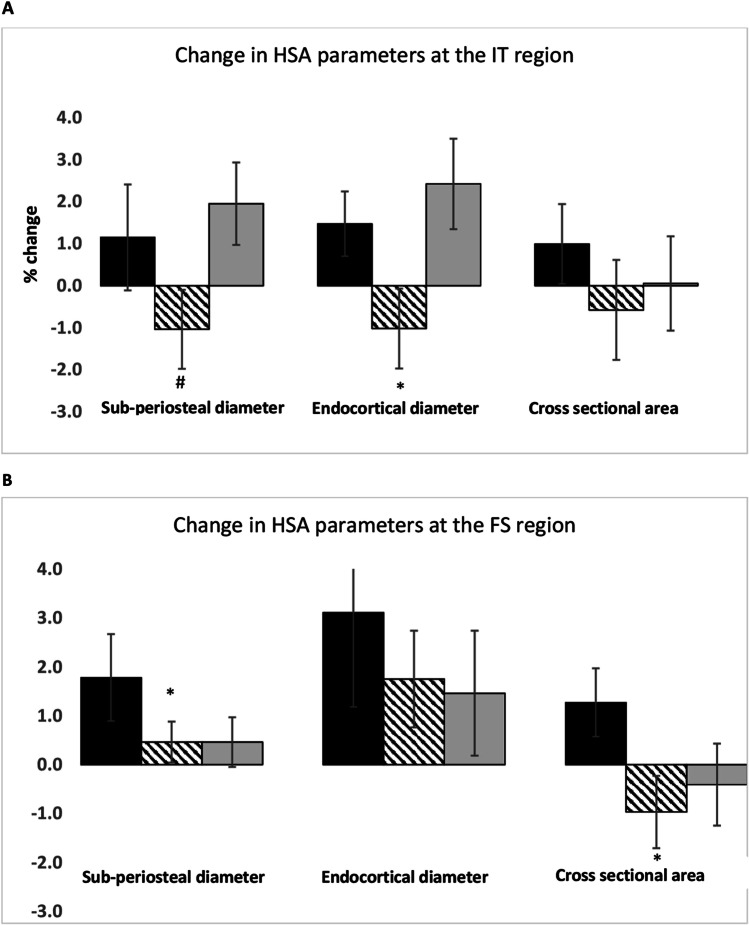
% Change in HSA parameters at the intertrochanteric (**A**) and femoral shaft (**B**) regions placebo:  , Vitamin K_1_ arm:  , MK-4 arm:  , * *p* < 0.05 compared to placebo, ^#^ *p* = 0.06 corrected for covariates in exploratory analyses. Results are expressed as mean [SEM]

No significant post-treatment differences were seen in the hip structural and mechanical parameters between the 3 arms at any sites; NN, IT or FS following ITT analysis. Following correction for covariates, there was a significant difference between change in endocortical diameter (ED) at the IT in the vitamin K_1_ arm compared to placebo. We observed a significant interaction between baseline vitamin K_1_ and the ED *(p* = *0.004)* independently of the treatment arms (Supplementary Table 2). In PP analyses, we found significant differences between placebo and vitamin K_1_ arm at the IT and FS sites, expressed as % change compared to placebo and following adjustment for covariates (Fig. 3 and Supplementary Table 2). We did not find any differences in the hip mechanical parameters (CSMI, Z and BR) between the 3 arms (Supplementary Tables 3 and 4).

### Safety

There were 11 serious adverse events (SAEs) and 245 reported AEs. Only 2 were possibly related to the IMP (reported diarrhoea and dizziness). Overall, 75 patients (71.4%) patients experienced at least one AE during the study period. The proportion that experienced at least one SAE was 7.6% (*n* = 8). There were no significant differences between the groups regarding the AEs and SAEs. There were no adverse drug reactions (ADRs), no Serious Adverse Reactions (SARs), no unexpected SARs and no SUSARs.

Twelve fractures occurred in 11 patients during the study, placebo *n* = 6, active vitamin K_1_ *n* = 4, active vitamin K_2_ (MK-4) *n* = 2. The number of fractures in each arm was small and the study was not powered to detect incident fractures as outcomes.

## Discussion

The purpose of this RCT was to determine whether treatment with bisphosphonates plus calcium and/or vitamin D combined with vitamin K, in post-menopausal women with serum vitamin K_1_ below the median for the cohort and osteoporosis would offer additional benefits on BMD, bone turnover and parameters of hip geometry and thus contribute to further reduction in fracture risk. The combination of vitamin K_1_ or MK-4 and oral bisphosphonate did not lead to significant improvement in BMD at the LS, TH and FN or any changes in bone turnover as assessed by CTX and P1NP. There were significant changes in hip structural parameters in particular the endocortical diameter (ED) at the inter-trochanter (IT) region and cross-sectional area at the femoral shaft (FS) in the vitamin K_1_ arm compared to placebo, suggesting that there may be potential synergism between vitamin K_1_ and bisphosphonates on hip geometry.

The effect of vitamin K supplementation on BMD has yielded mixed results as shown by a meta-analysis of 22 trials of vitamin K_1_ and K_2_ (MK-4 and MK-7) [13]. Modest synergy in terms of bone loss prevention has been shown in older women (> 60 years) at the ultra-distal radius only but not at other skeletal sites with a nutritionally achievable dose of 200 µg/day vitamin K_1_ and calcium plus vitamin D supplements at 18 and 24 months [21]. Another trial in healthy post-menopausal women showed no effect of vitamin K_1_ (1 mg/day) or MK-4 (45 mg/day) with calcium plus vitamin D supplements for 12 months on BMD [17]. Trials of MK-7 (another form of vitamin K_2_) have also shown discrepant results [22, 23]. A synergistic effect of bisphosphonates and vitamin K supplementation in osteoporosis has been proposed. A study of 231 patients with osteoporosis treated with either alendronate or risedronate showed that undercarboxylated osteocalcin (ucOC) was significantly higher in patients who sustained an incident fracture despite bisphosphonate [16]. The beneficial effects of combined MK-4 and alendronate therapy on serum ucOC concentrations and femoral neck BMD in PMO has been demonstrated in a small RCT, although treatment with alendronate was started at study entry [15]. However, a large study from Japan investigating the efficacy of combined MK4 and risedronate versus risedronate alone in PMO did not show any additional benefits of combined treatment on fracture and BMD compared to risedronate alone [24]. This would be consistent with our findings as we did not observe any additive effect of MK-4 supplementation in women with osteoporosis on established treatment with oral bisphosphonate. We observed a positive trend of 1% improvement in BMD at the hip in the vitamin K_1_ arm compared to placebo, although the results failed to reach significance. One explanation for our findings may be the duration of exposure to oral bisphosphonates as this could have affected the BMD response as a longer duration of treatment with vitamin K may be required to boost mineralisation and BMD due to the slower bone remodelling rate with bisphosphonates. Another confounding factor is that serum vitamin K_1_ concentrations were higher than 0.4 µg/L in a proportion of patients, particularly in the vitamin K_1_ arm at study entry, although the pre-screen values met the study entry criteria.

It has been suggested that vitamin K can affect other parameters of bone quality including bone turnover, mineralisation and collagen status. However previous studies of vitamin K_1_, MK-4 and MK-7 have failed to show any effect on BTMs including CTX and P1NP and a marker of mineralisation such as bone-specific alkaline phosphatase (Bone ALP) [17, 22, 23]. In a previous trial of alendronate alone or with MK-4, only changes in ucOC were observed in the MK4 arm but no between group differences were seen in BALP or deoxypyridinoline (DPD) [15]. In our trial, we did not see any group differences in CTX and P1NP, although we observed a gradual increase in the 2 markers over time which was similar in all 3 arms. This could have been due to concordance issues, although participants were asked if they were still taking oral bisphosphonate at each study visit, we did not do a pill count to formally assess adherence in all women as the bisphosphonates were prescribed in primary care. Studies report decreasing concordance with oral bisphosphonates with long-term use [25].

It has been postulated that vitamin K may have an effect on other parameters of bone strength including bone microarchitecture, geometry and strength, independently of BMD [26]. This has been shown in previous trials of MK-4 and MK-7, although in the latter trial, this effect was seen after 12 months treatment only [22, 23, 26]. Results of HSA parameters from our exploratory analyses show significant changes at the inter-trochanteric (IT) and femoral shaft (FS) areas of the hip in the vitamin K_1_ arm compared to placebo. In ageing, adaptive periosteal expansion occurs and with endocortical resorption, this leads to widening of both periosteal and endocortical diameters and greater cross-sectional area resulting in wider bones and a more peripheral distribution of bone material [27]. The addition of vitamin K_1_ to bisphosphonates in the treatment of osteoporosis may help further reduce these age-related changes and cortical thinning. The observed reduction in sub-periosteal diameter and endocortical diameter (ED) was similar to changes observed in trials of denosumab where HSA changes have been shown to be larger than with oral bisphosphonates as denosumab penetrates deeper into cortical bone [28–30]. This suggests a synergistic effect of vitamin K_1_ and bisphosphonate at the IT region in reducing endocortical widening, cortical thinning and improving structural stability. Biologically, this may be related, in part, to increased carboxylation of osteocalcin (OC) which has increased affinity for calcium and may thus lead to improved bone material properties. Although we did not measure ucOC in our study, the serum concentrations of vitamin K_1_ achieved in the vitamin K_1_ arm would suggest that the non-hepatic vitamin K binding proteins (VKDPs) including OC were maximally carboxylated [11, 18].

At the FS, we observed a significant reduction in the sub-periosteal diameter (OD) and cross sectional area (CSA) in the vitamin K_1_ arm only compared to placebo. The observed effects of combined treatment with vitamin K_1_ and bisphosphonate at the femoral shaft (FS) are of interest and should be interpreted in the context of duration of treatment with bisphosphonates which can alter cortical bone geometry and properties at this site due to long term suppression of bone remodelling [31]. This can increase the risk of atypical femoral fractures (AFF), a fatigue fracture rather than a traumatic fracture with specific features including the presence of localized periosteal or endosteal thickening of the lateral cortex as well as generalised cortical diaphyseal thickening [32]. Biomechanical factors of femur geometry have been implicated in the pathogenesis of AFF. Differences in femur geometry including wider femoral shaft diameter has been linked to the development of AFF at the shaft [33]. Thus, the observed reduction in OD and CSA suggests that addition of vitamin K_1_ to bisphosphonate may prevent cortical thickening at this site and reduce susceptibility to damage accumulation. This may protect against the risk of shaft AFF, particularly in patients on long term bisphosphonates. Further studies should investigate the vitamin K status of patients with AFF and whether vitamin K_1_ supplementation can prevent the development of AFF in patients on anti-resorptive agents.

We did not find any significant changes with MK-4 in any of the HSA parameters although MK-4 is thought to have specific functions on bone cells other than γ-carboxylation of VKDPs [34, 35], although the direct effects on bone cells may have been somewhat impaired due to suppression of bone remodelling by long term bisphosphonate therapy. The lack of a synergistic effect with bisphosphonates may be due, partly, to the poor bioavailability of MK-4 as the degree of absorption is dependent on dietary fat content with peak bioavailability seen when the lipid content of the meal is greater than 35 g [36]. MK-4 also has a short serum half—life compared to the other vitamin K_2_ series in particular MK-7 as consecutive MK-4 supplementation did not lead to increases of plasma MK-4 levels [37]. However, this study looked at nutritional doses of MK-4, whereas we used pharmacological doses. Although the same dose of MK-4 was used in previous studies, it was a different preparation from EISAI Co, Tokyo, Japan which was difficult to obtain for our study and which may be better absorbed than the preparation we used. We did not measure MK-4 in the circulation in our study because of a lack of established assays with adequate sensitivity.

The strength of our study is the design which was a double blind randomised controlled trial of post-menopausal women with osteoporosis. The participants had good compliance with the vitamin K supplements. Participants were seen frequently during the study and engaged with the trial. However, there were several limitations including the small size, short duration (18 months) as it may take longer for changes in BMD and/or the geometric/mechanical parameters to be seen, particularly at cortical rich sites in patients established on bisphosphonates. Not all patients had a low serum vitamin K_1_ at entry into the trial which could have reduced the treatment effects. However, this may help with the generalisability of the trial. We did not measure MK4 in serum and therefore we were unable to determine its bioavailability. The HSA software estimate of hip geometry assumes circular annuli of the femoral neck and shaft which may not be perfectly modelled. However, previous studies have shown that HSA parameters derived at the hip correlated highly to high-resolution QCT [38].

In conclusion, we investigated whether the addition of vitamin K_1_ or MK-4 had a synergistic effect on BMD, bone turnover and hip geometry/strength in post-menopausal women on established bisphosphonate treatment with calcium /vitamin D for osteoporosis. We did not observe any significant difference in change in BMD and BTMs between the treatment arms of the trial and placebo suggesting that vitamin K may not have any synergism with bisphosphonates on BMD or bone turnover. However, other components of bone strength including hip geometry and bone size may be influenced by the addition of vitamin K_1_ to standard treatment with bisphosphonates. Further larger studies of longer duration are needed to confirm this effect of vitamin K_1_ on hip geometry and in the prevention of AFF.

### Supplementary Information

Below is the link to the electronic supplementary material.

Supplementary file1 (DOCX 39 KB)

## Data Availability

The non-identifiable/anonymised data that support the findings of this study are available from the corresponding author, [GH], upon reasonable request by clinical or academic researchers for non-commercial use.
