# Supplementary material for: The additive effect of vitamin K supplementation and bisphosphonate on fracture risk in post-menopausal osteoporosis: a randomised placebo controlled trial
Source: Arch Osteoporos. 2023 Jun 20;18(1):83. doi: 10.1007/s11657-023-01288-w (PMC10282078; doi:10.1007/s11657-023-01288-w)
Supplement: Supplementary file 1 — Supplementary file1 (DOCX 39 KB) [file 11657_2023_1288_MOESM1_ESM.docx]

**Supplementary Table 1.** Changes in serum vitamin K_1_ at different time-points during the clinical trial. Significant differences were seen in the vitamin K_1_ arm compared to MK-4 and placebo *(p<0.001*).*

| Serum vitamin K_1_ conc. µg/L  mean (SD)  Timepoints | Vitamin K_1_  n=32 | 95% CI | MK-4  n=31 | 95% CI | Placebo  n=30 | 95% CI | *p-value* |
| --- | --- | --- | --- | --- | --- | --- | --- |
| 3 month | 3.12 (1.60) | 2.5, 3.7 | 0.43 (0.32) | 0.31, 0.54 | 0.46 (0.30) | 0.35, 0.57 | *<0.001** |
| 6 month | 3.23 (2.38) | 2.4, 4.1 | 0.48 (0.63) | 0.24, 0.71 | 0.52 (0.39) | 0.37, 0.66 | *<0.001** |
| 12 month | 3.08 (3.09) | 2.0, 4.2 | 0.36 (0.20) | 0.29, 0.43 | 0.45 (0.32) | 0.33, 0.56 | *<0.001** |
| 18 month | 2.56 (2.60) | 1.6, 3.5 | 0.40 (0.31) | 0.28, 0.51 | 0.59 (0.43) | 0.43, 0.75 | *<0.001** |

**Supplementary Table 2.** Significant changes in some hip structural analysis (HSA) parameters at the intertrochanter (IT) and femoral shaft (FS) following correction for co-variates age, BMI, baseline serum vitamin K_1_ concentration, duration of treatment with bisphosphonate and type of bisphosphonate (alendronate or risedronate) in the vitamin K_1_ arm compared to placebo following ITT (A) and PP analysis (B).

**A. ITT analysis**

| **IT endocortical diameter (ED)** | Estimate | Std. Error | t value | P value |
| --- | --- | --- | --- | --- |
| Vitamin K_1_ arm | -0.1069 | 0.05496 | -1.946 | 0.0552 |
| MK-4 arm | -0.03244 | 0.05452 | -0.595 | 0.5536 |
| Baseline serum vitamin K_1_ | -0.248 | 0.08566 | -2.895 | 0.004883 |

**B. PP analysis**

| **IT endocortical diameter (ED)** | Estimate | Std. Error | t value | P value |
| --- | --- | --- | --- | --- |
| Vitamin K_1_ arm | -0.1149 | 0.05551 | -2.07 | 0.04189 |
| MK-4 arm | -0.02565 | 0.05682 | -0.4515 | 0.6529 |

| **FS sub-periosteal diameter** | Estimate | Std. Error | t value | P value |  |
| --- | --- | --- | --- | --- | --- |
| Vitamin K_1_ arm | -0.05698 | 0.02815 | -2.024 | 0.0465 |  |
| MK-4 arm | -0.04612 | 0.02878 | -1.602 | 0.1132 |  |
| **Femoral shaft (FS) CSA** | | Estimate | Std. Error | t value | P value |
| Vitamin K_1_ arm | | -0.08838 | 0.03971 | -2.225 | 0.02902 |
| MK-4 arm | | -0.05123 | 0.04047 | -1.266 | 0.2095 |

**Supplementary Table 3.** % Change in HSA parameters in intention to treat (ITT) analysis.

ns: not significant, *p=0.055* vitamin K_1_ arm compared to placebo in exploratory analyses following correction for co-variates.*

| HSA Parameters  **ITT analysis** | Placebo n=30 | | | Vitamin K_1_ n=30 | | | MK-4 n=31 | | | *P value* |
| --- | --- | --- | --- | --- | --- | --- | --- | --- | --- | --- |
| **Narrow Neck (NN)**  mean [SD] | Baseline | 18-months | % change | Baseline | 18 -months | % change | Baseline | 18- months | % change | ns |
| Sub=periosteal diameter (OD) | 3.52  (0.36) | 3.56  (0.37) | 1.44  (6.75) | 3.52  (0.37) | 3.51  (0.32) | 0.05  (6.31) | 3.43  (0.30) | 3.46  (0.29) | 1.19  (6.95) | ns |
| Endocortical diameter (ED) | 3.24  (0.38) | 3.28  (0.40) | 1.53  (7.89) | 3.25  (0.39) | 3.24  (0.33) | 0.10  (7.08) | 3.14  (0.33) | 3.17  (0.32) | 1.44  (8.21) | ns |
| CSA | 2.45  (0.30) | 2.48  (0.34) | 1.13  (5.78) | 2.42  (0.39) | 2.44  (0.41) | 0.81  (6.70) | 2.48  (0.43) | 2.51  (0.46) | 1.04  (3.61) | ns |
| CSMI | 2.25  (0.55) | 2.26  (0.49) | 0.88  (9.71) | 2.20  (0.63) | 2.21  (0.71) | -0.01  (8.58) | 2.26  (0.55) | 2.32  (0.60) | 2.92  (8.79) | ns |
| Z ‘section modulus’ | 1.15  (0.21) | 1.14  (0.21) | 0.00  (9.10) | 1.11  (0.27) | 1.13  (0.29) | 1.26  (7.84) | 1.19  (0.25) | 1.20  (0.26) | 0.76  (7.55) | ns |
| Cortical thickness | 0.14  (0.02) | 0.14  (0.02) | 0.08  (9.97) | 0.14  (0.02) | 0.14  (0.02) | 1.11  (7.65) | 0.15  (0.03) | 0.15  (0.03) | -0.95  (7.76) | ns |
| Buckling Ratio (BR) | 14.45  (3.14) | 14.72  (3.99) | 2.02  (15.86) | 14.89  (3.40) | 14.58  (2.87) | -0.71  (12.02) | 13.65  (3.45) | 13.96  (3.35) | 3.69  (15.85) | ns |
| **Intertrochanter (IT)**  mean [SD] |  |  |  |  |  |  |  |  |  |  |
| Sub=periosteal diameter (OD) | 5.40  (0.36) | 5.46  (0.40) | 1.10  (3.57) | 5.52  (0.46) | 5.47  (0.38) | -0.64  (4.95) | 5.33  (0.43) | 5.41  (0.39) | 1.59  (5.23) | ns |
| Endocortical diameter (ED) | 4.79  (0.39) | 4.85  (0.42) | 1.32  (4.11) | 4.89  (0.44) | 4.85  (0.36) | -0.63  (5.10) | 4.69  (0.44) | 4.77  (0.39) | 2.04  (5.72) | *p=0.055** |
| CSA | 3.87  (0.59) | 3.91  (0.61) | 0.92  (4.95) | 3.97  (0.80) | 3.96  (0.76) | 0.06  (7.03) | 4.05  (0.59) | 4.03  (0.61) | -0.39  (5.91) | ns |
| CSMI | 10.55  (2.42) | 10.85  (2.62) | 3.03  (8.66) | 10.85  (3.00) | 10.79  (2.76) | 0.67  (13.19) | 10.41  (2.48) | 10.62  (2.80) | 2.77  (14.87) | ns |
| Z ‘section modulus’ | 3.32  (0.65) | 3.39  (0.71) | 2.36  (8.46) | 3.42  (0.76) | 3.43  (0.74) | 0.84  (11.17) | 3.33  (0.66) | 3.35  (0.72) | 1.19  (12.88) | ns |
| Cortical thickness | 0.31  (0.05) | 0.30  (0.05) | -0.55  (7.10) | 0.32  (0.06) | 0.31  (0.06) | 0.07  (6.51) | 0.32  (0.06) | 0.32  (0.05) | -1.10  (6.33) | ns |
| Buckling Ratio (BR) | 10.62  (1.95) | 10.80  (2.07) | 1.94  (8.85) | 10.26  (1.72) | 10.30  (1.91) | 0.33  (6.41) | 9.95  (2.03) | 10.20  (2.11) | 2.82  (7.45) | ns |
| **Femoral Shaft (FS)**  Mean [SD] |  |  |  |  |  |  |  |  |  |  |
| Sub=periosteal diameter (OD) | 3.01  (0.23) | 3.06  (0.27) | 1.65  (5.25) | 2.96  (0.24) | 2.98  (0.22) | 0.64  (2.43) | 3.00  (0.24) | 3.02  (0.24) | 0.49  (2.59) | ns |
| Endocortical diameter (ED) | 2.10  (0.34) | 2.15  (0.38) | 2.76  (10.22) | 2.09  (0.33) | 2.11  (0.30) | 1.77  (5.12) | 2.10  (0.36) | 2.13  (0.34) | 1.62  (6.61) | ns |
| CSA | 3.59  (0.60) | 3.64  (0.61) | 1.32  (3.70) | 3.45  (0.52) | 3.43  (0.52) | -0.31  (4.99) | 3.55  (0.46) | 3.54  (0.50) | -0.38  (4.46) | ns |
| CSMI | 3.19  (0.81) | 3.28  (0.85) | 3.21  (7.60) | 2.99  (0.82) | 3.04  (0.80) | 1.88  (7.42) | 3.19  (0.73) | 3.22  (0.80) | 0.94  (7.55) | ns |
| Z ‘section modulus’ | 2.03  (0.42) | 2.05  (0.42) | 1.38  (5.86) | 1.93  (0.39) | 1.95  (0.40) | 0.85  (6.23) | 2.03  (0.36) | 2.03  (0.39) | -0.13  (6.88) | ns |
| Cortical thickness | 0.45  (0.09) | 0.45  (0.09) | -0.17  (6.47) | 0.44  (0.08) | 0.43  (0.08) | -1.22  (6.00) | 0.45  (0.09) | 0.44  (0.08) | -0.98  (7.26) | ns |
| Buckling Ratio (BR) | 3.61  (0.89) | 3.71  (1.02) | 3.20  (15.40) | 3.62  (0.85) | 3.70  (0.80) | 2.86  (8.12) | 3.62  (0.86) | 3.69  (0.86) | 2.56  (9.45) | ns |

**Supplementary Table 4.** % Change in HSA parameters in per protocol (PP) analysis.

ns: not significant, *p<0.05* vitamin K_1_ arm compared to placebo in exploratory analyses following correction for co-variates.*

**PP analysis**

| HSA Parameters | Placebo n=28 | | | Vitamin K_1_ n=28 | | | MK-4 n=28 | | | *P value* |
| --- | --- | --- | --- | --- | --- | --- | --- | --- | --- | --- |
| **Narrow Neck (NN)**  mean [SD] | Baseline | 18-months | % change | Baseline | 18 -months | % change | Baseline | 18- months | % change |  |
| Sub=periosteal diameter (OD) | 3.52  (0.36) | 3.56  (0.38) | 1.15  (6.68) | 3.51  (0.37) | 3.48  (0.30) | -0.55  (6.02) | 3.42  (0.26) | 3.47  (0.26) | 1.61  (7.19) | ns |
| Endocortical diameter (ED) | 3.24  (0.39) | 3.28  (0.41) | 1.21  (7.83) | 3.24  (0.39) | 3.20  (0.31) | -0.53  (6.86) | 3.13  (0.30) | 3.18  (0.29) | 1.99  (8.46) | ns |
| CSA | 2.45  (0.30) | 2.48  (0.35) | 0.93  (5.78) | 2.43  (0.38) | 2.43  (0.41) | -0.06  (5.42) | 2.50  (0.44) | 2.52  (0.48) | 0.77  (3.42) | ns |
| CSMI | 2.26  (0.56) | 2.26  (0.50) | 0.93  (9.88) | 2.15  (0.55) | 2.14  (0.58) | -0.48  (8.45) | 2.24  (0.49) | 2.32  (0.54) | 3.59  (8.45) | ns |
| Z ‘section modulus’ | 1.15  (0.21) | 1.15  (0.21) | 0.31  (9.11) | 1.10  (0.25) | 1.11  (0.26) | 1.04  (8.04) | 1.20  (0.26) | 1.21  (0.26) | 1.11  (7.52) | ns |
| Cortical thickness | 0.14  (0.02) | 0.14  (0.03) | 0.08  (10.15) | 0.14  (0.02) | 0.14  (0.02) | 1.11  (7.63) | 0.15  (0.03) | 0.15  (0.03) | -1.47  (7.79) | ns |
| Buckling Ratio (BR) | 14.52  (3.18) | 14.74  (4.06) | 1.57  (15.97) | 14.75  (3.46) | 14.43  (2.92) | -0.71  (12.17) | 13.41  (3.19) | 13.88  (3.17) | 4.92  (16.21) | ns |
| **Intertrochanter (IT)**  mean [SD] |  |  |  |  |  |  |  |  |  |  |
| Sub=periosteal diameter (OD) | 5.41  (0.37) | 5.47  (0.40) | 1.22  (3.57) | 5.53  (0.44) | 5.46  (0.35) | -1.04  (4.89) | 5.32  (0.45) | 5.41  (0.39) | 1.95  (5.19) | ns |
| Endocortical diameter (ED) | 4.80  (0.39) | 4.87  (0.41) | 1.47  (4.09) | 4.90  (0.43) | 4.83  (0.34) | -1.02  (5.07) | 4.67  (0.45) | 4.76  (0.38) | 2.42  (5.74) | *p=0.04** |
| CSA | 3.86  (0.59) | 3.90  (0.62) | 0.99  (5.03) | 3.98  (0.79) | 3.95  (0.77) | -0.58  (6.29) | 4.11  (0.58) | 4.10  (0.59) | -0.05  (5.93) | ns |
| CSMI | 10.54  (2.46) | 10.87  (2.67) | 3.29  (8.71) | 10.86  (2.94) | 10.74  (2.74) | -0.15  (12.94) | 10.60  (2.53) | 10.87  (2.77) | 3.59  (14.90) | ns |
| Z ‘section modulus’ | 3.31  (0.66) | 3.39  (0.72) | 2.54  (8.56) | 3.43  (0.76) | 3.42  (0.76) | 0.03  (10.62) | 3.40  (0.65) | 3.43  (0.70) | 1.60  (13.03) | ns |
| Cortical thickness | 0.30  (0.05) | 0.30  (0.05) | -0.67  (7.20) | 0.32  (0.06) | 0.32  (0.06) | -0.39  (6.28) | 0.33  (0.06) | 0.32  (0.05) | -0.84  (6.40) | ns |
| Buckling Ratio (BR) | 10.68  (1.96) | 10.88  (2.06) | 2.13  (8.95) | 10.19  (1.76) | 10.26  (1.97) | 0.67  (6.30) | 9.71  (1.70) | 9.95  (1.81) | 2.85  (7.78) | ns |
| **Femoral Shaft (FS)**  Mean [SD] |  |  |  |  |  |  |  |  |  |  |
| Sub=periosteal diameter (OD) | 3.01  (0.23) | 3.06  (0.27) | 1.78  (5.30) | 2.95  (0.23) | 2.96  (0.21) | 0.46  (2.23) | 3.00  (0.21) | 3.01  (0.22) | 0.46  (2.71) | *p=0.04** |
| Endocortical diameter (ED) | 2.10  (0.34) | 2.16  (0.38) | 3.11  (10.23) | 2.07  (0.33) | 2.10  (0.30) | 1.75  (5.25) | 2.09  (0.33) | 2.11  (0.31) | 1.46  (6.79) | ns |
| CSA | 3.58  (0.61) | 3.63  (0.62) | 1.28  (3.76) | 3.44  (0.53) | 3.41  (0.52) | -0.97  (3.91) | 3.59  (0.47) | 3.58  (0.51) | -0.41  (4.43) | *p=0.029** |
| CSMI | 3.20  (0.82) | 3.30  (0.87) | 3.44  (7.64) | 2.94  (0.77) | 2.95  (0.74) | 0.87  (4.80) | 3.22  (0.71) | 3.24  (0.77) | 0.60  (7.82) | ns |
| Z ‘section modulus’ | 2.03  (0.43) | 2.05  (0.43) | 1.47  (5.94) | 1.91  (0.38) | 1.91  (0.39) | -0.01  (4.39) | 2.06  (0.36) | 2.05  (0.39) | -0.33  (6.99) | ns |
| Cortical thickness | 0.45  (0.09) | 0.45  (0.09) | -0.40  (6.47) | 0.44  (0.08) | 0.43  (0.08) | -1.67  (5.81) | 0.46  (0.09) | 0.45  (0.08) | -0.84  (7.37) | ns |
| Buckling Ratio (BR) | 3.63  (0.89) | 3.74  (1.03) | 3.55  (15.56) | 3.59  (0.86) | 3.69  (0.82) | 3.24  (8.28) | 3.56  (0.77) | 3.63  (0.82) | 2.38  (9.46) | ns |
